# Crystal structure of bis­(μ-3-nitro­benzoato)-κ^3^ *O*,*O*′:*O*;κ^3^ *O*:*O*,*O*′-bis­[bis­(3-cyano­pyridine-κ*N* ^1^)(3-nitro­benzoato-κ^2^ *O*,*O*′)cadmium]

**DOI:** 10.1107/S2056989017002675

**Published:** 2017-02-21

**Authors:** Tuncer Hökelek, Nurcan Akduran, Azer Özen, Güventürk Uğurlu, Hacali Necefoğlu

**Affiliations:** a Department of Physics, Hacettepe University, 06800 Beytepe, Ankara, Turkey; b SANAEM, Saray Mahallesi, Atom Caddesi, No. 27, 06980 Saray-Kazan, Ankara, Turkey; c Department of Chemistry, Kafkas University, 36100 Kars, Turkey; d International Scientific Research Centre, Baku State University, 1148 Baku, Azerbaijan

**Keywords:** crystal structure, cadmium, transition metal complex, benzoic acid

## Abstract

The asymmetric unit of the title compound, [Cd_2_(C_7_H_4_NO_4_)_4_(C_6_H_4_N_2_)_4_], contains one Cd^II^ atom, two 3-nitro­benzoate (NB) anions and two 3-cyano­pyridine (CPy) ligands. The two CPy ligands act as monodentate N(pyridine)-bonding ligands, while the two NB anions act as bidentate ligands through the carboxyl­ate O atoms. The centrosymmetric dinuclear complex is generated by application of inversion symmetry, whereby the Cd^II^ atoms are bridged by the carboxyl­ate O atoms of two symmetry-related NB anions, thus completing the distorted N_2_O_5_ penta­gonal–bipyramidal coordination sphere of each Cd^II^ atom. The benzene and pyridine rings are oriented at dihedral angles of 10.02 (7) and 5.76 (9)°, respectively. In the crystal, C—H⋯N hydrogen bonds link the mol­ecules, enclosing *R*_2_^2^(26) ring motifs, in which they are further linked *via* C—H⋯O hydrogen bonds, resulting in a three-dimensional network. In addition, π–π stacking inter­actions between parallel benzene rings and between parallel pyridine rings of adjacent mol­ecules [shortest centroid-to-centroid distances = 3.885 (1) and 3.712 (1) Å, respectively], as well as a weak C—H⋯π inter­action, may further stabilize the crystal structure.

## Abstract 2

In the title cadmium complex of 3-nitro­benzoate and 3-cyano­pyridine, binuclear centrosymmetric mol­ecules are present, with cadmium being surrounded in an N_2_O_5_ coordinaton set in a distorted penta­gonal–bipyramidal shape.

## Chemical context   

In the last two decades, research on metal–organic frameworks (MOFs) has received considerable attention due to their extensive structural chemistry (Li *et al.*, 2016 ▸) and their potential applications, including gas storage, nonlinear optics and ion exchange (Carlucci *et al.*, 2003 ▸). In the syntheses of compounds having MOF structures, various carboxyl­ate ligands have been used (Li *et al.*, 2004 ▸).
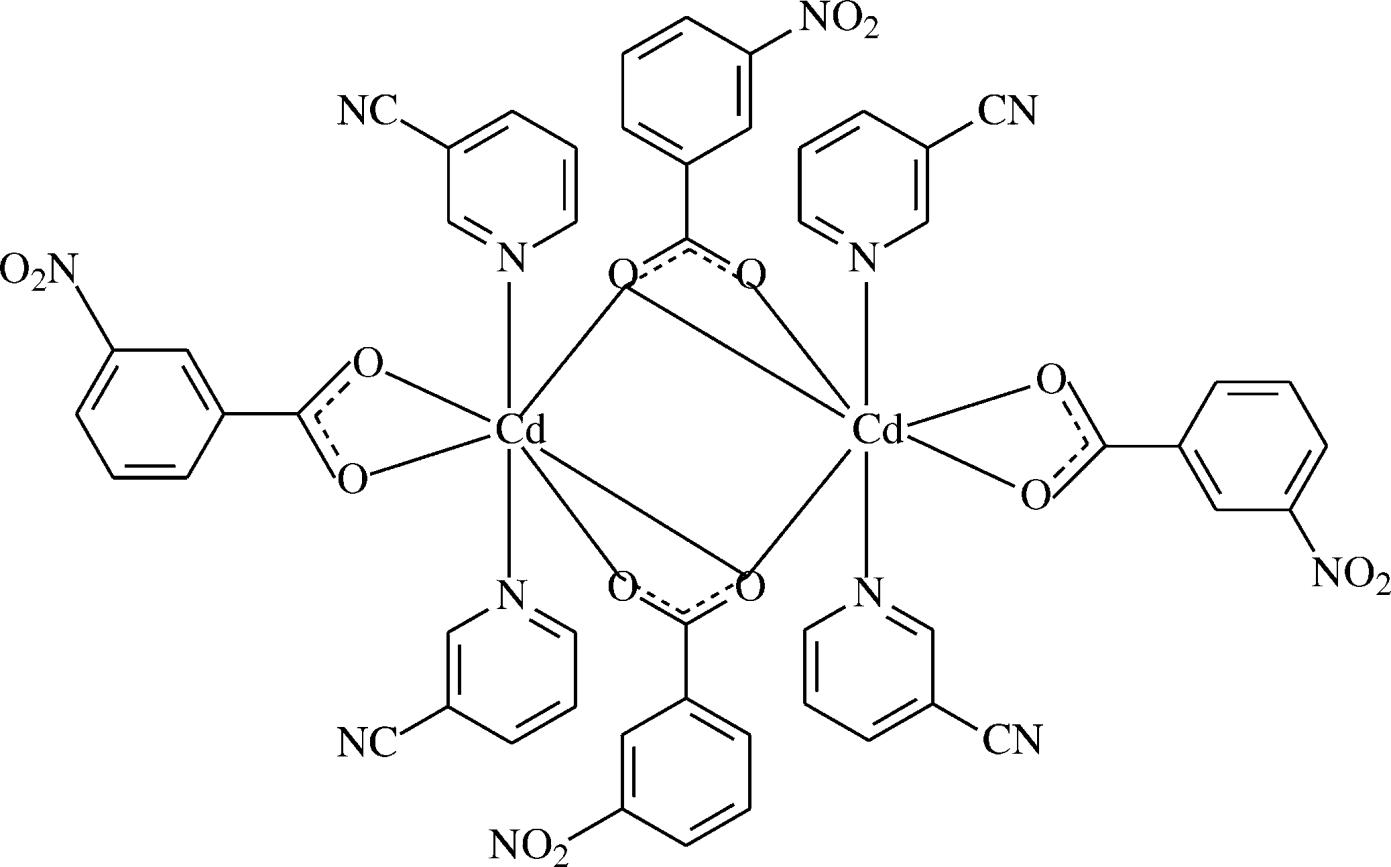


On the other hand, transition-metal complexes with biochemically active mol­ecules show inter­esting physical and/or chemical properties, through which they may find applications in biological systems (Antolini *et al.*, 1982 ▸). Some benzoic acid derivatives, such as 4-amino­benzoic acid, have been extensively reported in coordination chemistry, as bifunctional organic ligands, due to the varieties of their coordination modes (Chen & Chen, 2002 ▸; Amiraslanov *et al.*, 1979 ▸; Hauptmann *et al.*, 2000 ▸).

The structure–function–coordination relationships of aryl­carboxyl­ate ions in Zn^II^ complexes of benzoic acid derivatives change depending on the nature and position of the substituted groups on the benzene ring, the nature of the additional ligand mol­ecule or solvent, and the pH range and temperature of the synthesis (Shnulin *et al.*, 1981 ▸; Nadzhafov *et al.*, 1981 ▸; Antsyshkina *et al.*, 1980 ▸; Adiwidjaja *et al.*, 1978 ▸). When pyridine and its derivatives are used instead of water mol­ecules, the structure is completely different (Catterick *et al.*, 1974 ▸).

The structures of some dinuclear complexes obtained from the reactions of transition metal(II) ions with nicotinamide (NA; C_6_H_6_N_2_O) and some benzoic acid derivatives as ligands, *e.g.* [Zn_2_(C_7_H_4_FO_2_)_4_(C_6_H_6_N_2_O)_2_]·C_7_H_5_FO_2_ [(II); Hökelek *et al.*, 2009 ▸], [Cu_2_(C_8_H_7_O_3_)_4_(C_6_H_6_N_2_O)_2_(H_2_O)_2_] [(III); Hökelek *et al.*, 2010 ▸], [Cu_2_(C_8_H_5_O_3_)_4_(C_6_H_6_N_2_O)_4_] [(IV); Sertçelik *et al.*, 2013 ▸], [Mn_2_(C_7_H_4_BrO_2_)_4_(C_6_H_6_N_2_O)_2_(H_2_O)_2_] [(V); Necefoğlu *et al.*, 2011 ▸] and [Cd_2_(C_7_H_4_ClO_2_)_4_(C_6_H_6_N_2_O)_2_(H_2_O)_2_] [(VI); Bozkurt *et al.*, 2013 ▸], have been determined previously. In this context, we have synthesized the Cd^II^-containing title compound, bis(μ-3-nitro­benzoato)-κ^3^ *O*,*O*′:*O*;κ^3^ *O*:*O*,*O*′-bis­[bis­(3-cyano­pyridine-κ*N*)(3-nitro­benzoato-κ^2^ *O*,*O*′)cadmium], [Cd_2_(C_7_H_4_NO_4_)_4_(C_6_H_4_N_2_)_4_], and report herein its crystal structure.

## Structural commentary   

**Figure 1:**
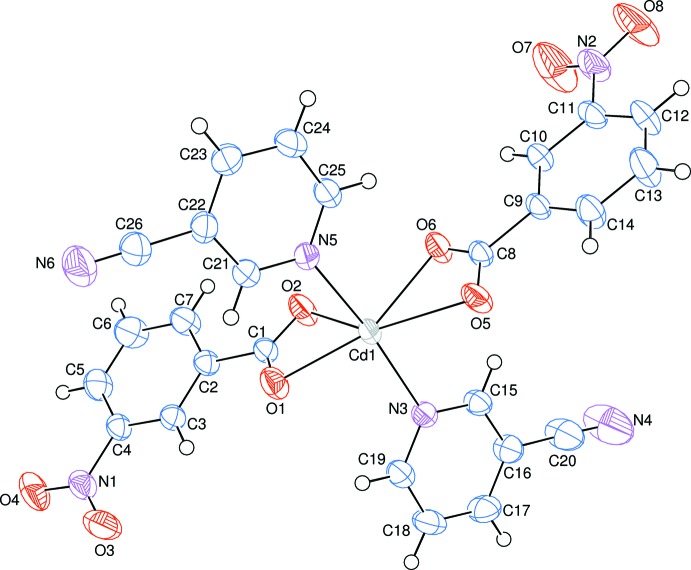
The asymmetric unit of the title mol­ecule, showing the atom-numbering scheme. Displacement ellipsoids are drawn at the 50% probability level.

**Figure 2:**
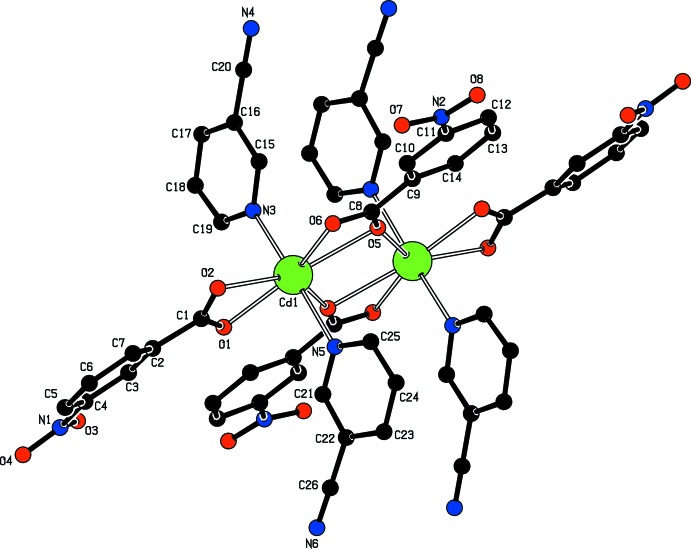
The mol­ecular structure of the binuclear title mol­ecule. Symmetry-related atoms are related by the symmetry code (−*x*, −*y* + 1, −*z* + 1). H atoms have been omitted for clarity.

The asymmetric unit of the title complex contains one Cd^II^ atom, two 3-nitro­benzoate (NB) anions and two 3-cyano­pyridine (CPy) ligands. The two CPy ligands are monodentate (through the pyridine N atoms), while both NB anions act as bidentate ligands through their carboxyl­ate O atoms (Fig. 1 ▸). The centrosymmetric dinuclear mol­ecule is completed by application of inversion symmerty. The Cd^II^ atoms are bridged by the carboxyl­ate O atoms of one NB anions (O6 and O5) and its symmetry-related counterpart [symmetry code: (i) −*x*, −*y* + 1, −*z* + 1]. Hence, this carboxyl­ate group not only chelates to one Cd^II^ atom but also bridges two Cd^II^ atoms (Fig. 2 ▸). Thus, each Cd^II^ atom is surrounded by three NB anions and two CPy ligands. The overall coordination sphere of the Cd^II^ atom is defined by the bridging/chelating NB anions (O5, O5^i^ and O6), one chelating NB anion (O1 and O2) and two 3-cyano­pyridine (CPy) ligands (N3 and N5), resulting in a distorted penta­gonal–bipyramidal environment. The five carboxyl­ate O atoms (O1, O2, O5, O5^i^ and O6) of the three NB anions around the Cd^II^ atom form a distorted penta­gonal arrangement, with an average Cd1—O bond length of 2.42 Å (Table 1 ▸). The distorted penta­gonal–bipyramidal coordination is completed by pyridine atoms N3 and N5 of the CPy ligands at distances of 2.3186 (17) and 2.3435 (17) Å, respectively, in the axial positions (Table 1 ▸; Figs. 1 ▸ and 2 ▸). The Cd1 atom lies 0.1252 (1) Å above and 0.0326 (1) Å below of planar O1/O2/C1 and O5/O6/C8 carboxyl­ate groups, respectively. The Cd1⋯Cd1^i^ separation in the binuclear mol­ecule is 3.9360 (15) Å and is comparable to the corresponding *M*—*M* distances (*M* is a metal) in the structurally related transition metal(II) complexes [7.1368 (3) Å in (III), 4.1554 (8) Å in (IV), 7.180 (2) Å in (V) and 7.1647 (3) Å in (VI)]. The metal atoms are bridged by two NA ligand N and O atoms in (III), (V) and (VI), while they are bridged by two carboxyl­ate O atoms in (IV).

The near equalities of the C1—O1 [1.264 (3) Å], C1—O2 [1.241 (3) Å], C8—O5 [1.256 (3) Å] and C8—O6 [1.253 (3) Å] bonds in the carboxyl­ate groups indicate delocalized bonding arrangements, rather than localized single and double bonds. The O1—Cd1—O2 and O5—Cd1—O6 bite angles are reduced to 54.33 (5) and 53.47 (5)°, respectively. The corresponding O—*M*—O (*M* is a divalent metal) angles are 60.92 (12)° in (II), 53.50 (14)° in (IV), 57.61 (8)° in (V), and 54.22 (4) and 53.32 (5)° in (VI). The dihedral angles between the planar carboxyl­ate groups (O1/O2/C1 and O5/O6/C8) and the adjacent benzene [*A* (C2–C7) and *B* (C9–C14)] rings in the title structure are 17.18 (13) and 3.36 (12)°, respectively, while the benzene (*A* and *B*) and pyridine [*C* (N3/C15–C19) and *D* (N5/C21–C25)] rings are oriented at dihedral angles of *A*/*B* = 10.02 (7)°, *A*/*C* = 72.70 (7)°, *A*/*D* = 74.72 (7)°, *B*/*C* = 82.28 (7)°, *B*/*D* = 84.54 (8)° and *C*/*D* = 5.76 (9)°.

## Supra­molecular features   

**Figure 3:**
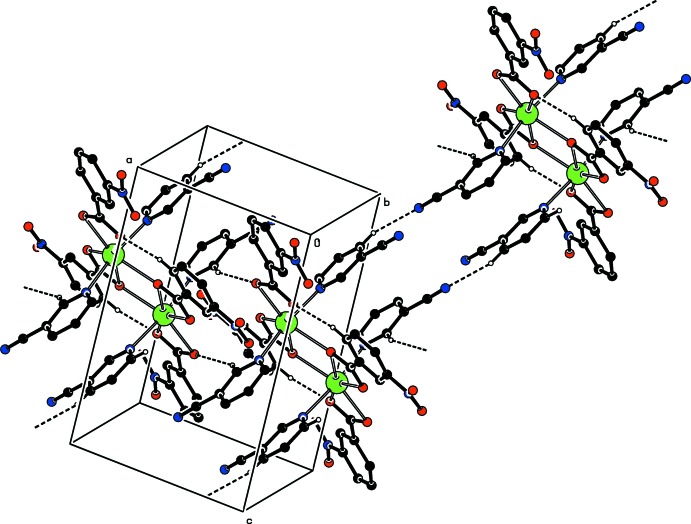
Part of the crystal structure. Intra­molecular (C—H_cpy_⋯O_c_ and C—H_nb_⋯O_c_) and inter­molecular (C—H_cpy_⋯N_cpy_ and C—H_cpy_⋯O_nb_) (cpy = cyano­pyridine, c = carboxyl­ate and nb = nitro­benzoate) hydrogen bonds are shown as dashed lines. Nonbonding H atoms have been omitted for clarity.

Intra­molecular C—H_cpy_⋯O_c_ (cpy = cyano­pyridine and c = carboxyl­ate) and C—H_nb_⋯O_c_ (nb = nitro­benzoate) hydrogen bonds (Table 2 ▸) link the cyano­pyridine and nitro­benzoate ligands to the carboxyl­ate O atoms. In the crystal, C—H_cpy_⋯N_cpy_ hydrogen bonds (Table 2 ▸) link the mol­ecules, enclosing 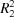
(26) ring motifs (Bernstein *et al.*, 1995 ▸) (Fig. 3 ▸), in which they are further linked *via* additional C—H_cpy_⋯O_nb_ (nb = nitro­benzoate) hydrogen bonds (Table 2 ▸), resulting in a three-dimensional network. The π–π contacts between parallel benzene rings and between parallel pyridine rings of adjacent mol­ecules, *Cg*1–*Cg*2^i^ and *Cg*3–*Cg*4^ii^ [symmetry codes: (i) −*x* + 1, −*y* + 1, −*z* + 1; (ii) −*x*, −*y* + 1, −*z* + 1; *Cg*1, *Cg*2, *Cg*3 and *Cg*4 are the centroids of the rings *A* (atoms C2–C7), *B* (C9–C14), *C* (N3/C15–C19) and *D* (N5/C21–C25)] may further stabilize the structure, with centroid–centroid distances of 3.885 (1) and 3.712 (1) Å, respectively. A weak C—H⋯π inter­action (Table 2 ▸) is also observed.

## Refinement   

The experimental details including the crystal data, data collection and refinement are summarized in Table 3 ▸. Aromatic H atoms were positioned geometrically, with C—H = 0.93 Å, and constrained to ride on their parent atoms, with *U*_iso_(H) = 1.2*U*_eq_(C). The maximum and minimum electron densities were found at 1.43 and 0.80 Å from atoms O2 and Cd1, respectively.

## Synthesis and crystallization   

The title compound was prepared by the reaction of 3CdSO_4_·8H_2_O (0.64 g, 2.5 mmol) in H_2_O (50 ml) and 3-cyano­pyridine (0.52 g, 5 mmol) in H_2_O (50 ml) with sodium 3-nitro­benzoate (0.95 g, 5 mmol) in H_2_O (100 ml) at 333 K. The mixture was filtered and set aside to crystallize at ambient temperature for one week, giving colourless single crystals.

## Supplementary Material

Crystal structure: contains datablock(s) I, global. DOI: 10.1107/S2056989017002675/wm5366sup1.cif

Structure factors: contains datablock(s) I. DOI: 10.1107/S2056989017002675/wm5366Isup2.hkl

CCDC reference: 1533101

Additional supporting information:  crystallographic information; 3D view; checkCIF report

## Figures and Tables

**Table 1:** Selected bond lengths (Å)

Cd1—O1	2.3017 (15)	Cd1—O6	2.3264 (15)
Cd1—O2	2.5072 (18)	Cd1—N3	2.3186 (17)
Cd1—O5	2.5367 (16)	Cd1—N5	2.3435 (17)
Cd1—O5^i^	2.4716 (16)		

Symmetry code: (i) 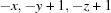.

**Table 2:** Hydrogen-bond geometry (Å, °) *Cg*3 is the centroid of the N3/C15–C19 ring.

*D*—H⋯*A*	*D*—H	H⋯*A*	*D*⋯*A*	*D*—H⋯*A*
C14—H14⋯O1^i^	0.93	2.20	3.108 (3)	167
C15—H15⋯O2^ii^	0.93	2.32	3.111 (3)	143
C23—H23⋯N4^iii^	0.93	2.38	3.236 (5)	154
C25—H25⋯O6	0.93	2.58	3.242 (3)	128
C10—H10⋯*Cg*3^ii^	0.93	3.26	4.186 (3)	176

Symmetry codes: (i) 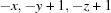; (ii) 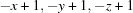; (iii) 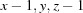.

**Table 3:** Experimental details

Crystal data	
Chemical formula	[Cd_2_(C_7_H_4_NO_4_)_4_(C_6_H_4_N_2_)_4_]
*M*_r_	1305.72
Crystal system, space group	Triclinic, *P* 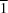
Temperature (K)	296
*a*, *b*, *c* (Å)	8.5237 (3), 12.7145 (4), 13.0583 (5)
α, β, γ (°)	105.022 (3), 97.347 (3), 104.866 (2)
*V* (Å^3^)	1292.12 (8)
*Z*	1
Radiation type	Mo *K*α
μ (mm^−1^)	0.91
Crystal size (mm)	0.28 × 0.20 × 0.18

Data collection	
Diffractometer	Bruker APEXII CCD
Absorption correction	Multi-scan (*SADABS*; Bruker, 2012 ▸)
*T*_min_, *T*_max_	0.615, 0.746
No. of measured, independent and observed [*I* > 2σ(*I*)] reflections	65471, 6414, 5828
*R*_int_	0.029
(sin θ/λ)_max_ (Å^−1^)	0.668

Refinement	
*R*[*F* ^2^ > 2σ(*F* ^2^)], *wR*(*F* ^2^), *S*	0.029, 0.072, 1.22
No. of reflections	6414
No. of parameters	370
H-atom treatment	H-atom parameters constrained
Δρ_max_, Δρ_min_ (e Å^−3^)	1.01, −0.75

Computer programs: *APEX2* (Bruker, 2012 ▸), *SAINT* (Bruker, 2012 ▸), *SHELXS97* (Sheldrick, 2008 ▸), *SHELXL97* (Sheldrick, 2008 ▸), *ORTEP-3* for Windows (Farrugia, 2012 ▸), *WinGX* (Farrugia, 2012 ▸) and *PLATON* (Spek, 2009 ▸).

## supplementary crystallographic information

### Crystal data

**Table:**

[Cd_2_(C_7_H_4_NO_4_)_4_(C_6_H_4_N_2_)_4_]	*Z* = 1
*M**_r_* = 1305.72	*F*(000) = 652
Triclinic, *P*1	*D*_x_ = 1.678 Mg m^−^^3^
Hall symbol: -P 1	Mo *K*α radiation, λ = 0.71073 Å
*a* = 8.5237 (3) Å	Cell parameters from 9607 reflections
*b* = 12.7145 (4) Å	θ = 3.1–28.3°
*c* = 13.0583 (5) Å	µ = 0.91 mm^−^^1^
α = 105.022 (3)°	*T* = 296 K
β = 97.347 (3)°	Prism, colourless
γ = 104.866 (2)°	0.28 × 0.20 × 0.18 mm
*V* = 1292.12 (8) Å^3^	

### Data collection

**Table:**

Bruker APEXII CCD diffractometer	6414 independent reflections
Radiation source: fine-focus sealed tube	5828 reflections with *I* > 2σ(*I*)
Graphite monochromator	*R*_int_ = 0.029
φ and ω scans	θ_max_ = 28.3°, θ_min_ = 3.1°
Absorption correction: multi-scan (*SADABS*; Bruker, 2012)	*h* = −11→11
*T*_min_ = 0.615, *T*_max_ = 0.746	*k* = −16→16
65471 measured reflections	*l* = −17→17

### Refinement

**Table:**

Refinement on *F*^2^	Primary atom site location: structure-invariant direct methods
Least-squares matrix: full	Secondary atom site location: difference Fourier map
*R*[*F*^2^ > 2σ(*F*^2^)] = 0.029	Hydrogen site location: inferred from neighbouring sites
*wR*(*F*^2^) = 0.072	H-atom parameters constrained
*S* = 1.22	*w* = 1/[σ^2^(*F*_o_^2^) + (0.0277*P*)^2^ + 0.8651*P*] where *P* = (*F*_o_^2^ + 2*F*_c_^2^)/3
6414 reflections	(Δ/σ)_max_ = 0.001
370 parameters	Δρ_max_ = 1.01 e Å^−^^3^
0 restraints	Δρ_min_ = −0.75 e Å^−^^3^

### Special details

**Table:**

Geometry. All esds (except the esd in the dihedral angle between two l.s. planes) are estimated using the full covariance matrix. The cell esds are taken into account individually in the estimation of esds in distances, angles and torsion angles; correlations between esds in cell parameters are only used when they are defined by crystal symmetry. An approximate (isotropic) treatment of cell esds is used for estimating esds involving l.s. planes.
Refinement. Refinement of F^2^ against ALL reflections. The weighted R-factor wR and goodness of fit S are based on F^2^, conventional R-factors R are based on F, with F set to zero for negative F^2^. The threshold expression of F^2^ > 2sigma(F^2^) is used only for calculating R-factors(gt) etc. and is not relevant to the choice of reflections for refinement. R-factors based on F^2^ are statistically about twice as large as those based on F, and R- factors based on ALL data will be even larger.

### Fractional atomic coordinates and isotropic or equivalent isotropic displacement parameters (Å^2^)

**Table:**

	*x*	*y*	*z*	*U*_iso_*/*U*_eq_	
Cd1	0.151839 (18)	0.436274 (11)	0.419807 (11)	0.03200 (6)	
O1	0.0821 (2)	0.25149 (13)	0.30752 (14)	0.0448 (4)	
O2	0.3355 (2)	0.35240 (13)	0.30991 (15)	0.0480 (4)	
O3	−0.0992 (3)	−0.15593 (16)	0.12938 (19)	0.0675 (6)	
O4	0.0033 (3)	−0.22812 (15)	−0.00392 (17)	0.0646 (5)	
O5	0.1364 (2)	0.61626 (13)	0.55320 (14)	0.0431 (4)	
O6	0.3515 (2)	0.61460 (13)	0.47720 (13)	0.0414 (3)	
O7	0.8058 (3)	0.9744 (2)	0.5573 (3)	0.1093 (12)	
O8	0.7807 (3)	1.13029 (18)	0.6485 (3)	0.0928 (9)	
N1	0.0007 (3)	−0.14806 (16)	0.07056 (17)	0.0435 (4)	
N2	0.7303 (3)	1.02738 (18)	0.6099 (2)	0.0537 (5)	
N3	0.2275 (2)	0.37248 (14)	0.56414 (14)	0.0320 (3)	
N4	0.6329 (5)	0.5371 (3)	0.8932 (3)	0.1256 (18)	
N5	0.0320 (2)	0.48389 (15)	0.27322 (14)	0.0358 (4)	
N6	−0.3803 (3)	0.2705 (2)	−0.0491 (2)	0.0685 (7)	
C1	0.2174 (3)	0.26302 (16)	0.27548 (16)	0.0324 (4)	
C2	0.2308 (3)	0.16188 (17)	0.19015 (17)	0.0322 (4)	
C3	0.1160 (3)	0.05585 (17)	0.17155 (17)	0.0330 (4)	
H3	0.0350	0.0469	0.2127	0.040*	
C4	0.1244 (3)	−0.03626 (17)	0.09058 (17)	0.0347 (4)	
C5	0.2408 (3)	−0.0262 (2)	0.0267 (2)	0.0459 (5)	
H5	0.2429	−0.0892	−0.0279	0.055*	
C6	0.3544 (4)	0.0798 (2)	0.0456 (2)	0.0535 (6)	
H6	0.4340	0.0886	0.0034	0.064*	
C7	0.3498 (3)	0.1736 (2)	0.1279 (2)	0.0447 (5)	
H7	0.4274	0.2445	0.1410	0.054*	
C8	0.2760 (2)	0.66594 (16)	0.53913 (16)	0.0315 (4)	
C9	0.3550 (2)	0.79211 (16)	0.59657 (16)	0.0312 (4)	
C10	0.5053 (3)	0.84920 (17)	0.57676 (18)	0.0346 (4)	
H10	0.5592	0.8106	0.5294	0.042*	
C11	0.5721 (3)	0.96539 (17)	0.62989 (19)	0.0384 (5)	
C12	0.4989 (3)	1.02590 (19)	0.7024 (2)	0.0491 (6)	
H12	0.5479	1.1036	0.7373	0.059*	
C13	0.3506 (4)	0.9677 (2)	0.7217 (2)	0.0565 (7)	
H13	0.2988	1.0064	0.7706	0.068*	
C14	0.2785 (3)	0.8520 (2)	0.6685 (2)	0.0453 (5)	
H14	0.1776	0.8140	0.6811	0.054*	
C15	0.3517 (3)	0.43881 (18)	0.64654 (18)	0.0360 (4)	
H15	0.4061	0.5123	0.6466	0.043*	
C16	0.4023 (3)	0.4017 (2)	0.73219 (19)	0.0414 (5)	
C17	0.3240 (3)	0.2918 (2)	0.7327 (2)	0.0479 (6)	
H17	0.3567	0.2653	0.7894	0.058*	
C18	0.1962 (3)	0.2229 (2)	0.6465 (2)	0.0503 (6)	
H18	0.1411	0.1486	0.6437	0.060*	
C19	0.1517 (3)	0.26648 (19)	0.56478 (19)	0.0417 (5)	
H19	0.0649	0.2200	0.5072	0.050*	
C20	0.5330 (4)	0.4779 (3)	0.8215 (3)	0.0706 (10)	
C21	−0.0872 (3)	0.40583 (18)	0.19319 (17)	0.0375 (4)	
H21	−0.1240	0.3318	0.1971	0.045*	
C22	−0.1584 (3)	0.43099 (19)	0.10448 (17)	0.0373 (4)	
C23	−0.1049 (3)	0.5416 (2)	0.09792 (19)	0.0462 (5)	
H23	−0.1512	0.5609	0.0397	0.055*	
C24	0.0186 (4)	0.6213 (2)	0.1803 (2)	0.0541 (7)	
H24	0.0579	0.6960	0.1787	0.065*	
C25	0.0835 (3)	0.58913 (19)	0.26552 (19)	0.0460 (6)	
H25	0.1675	0.6438	0.3204	0.055*	
C26	−0.2833 (3)	0.3418 (2)	0.0192 (2)	0.0484 (6)	

### Atomic displacement parameters (Å^2^)

**Table:**

	*U*^11^	*U*^22^	*U*^33^	*U*^12^	*U*^13^	*U*^23^
Cd1	0.03932 (9)	0.02142 (7)	0.02841 (8)	0.00423 (6)	0.00100 (6)	0.00389 (5)
O1	0.0451 (9)	0.0315 (8)	0.0489 (9)	0.0062 (7)	0.0178 (7)	−0.0009 (7)
O2	0.0433 (9)	0.0304 (8)	0.0549 (10)	−0.0010 (7)	0.0077 (8)	0.0003 (7)
O3	0.0779 (14)	0.0390 (10)	0.0778 (14)	0.0002 (9)	0.0284 (12)	0.0163 (10)
O4	0.0769 (14)	0.0324 (9)	0.0658 (13)	0.0110 (9)	0.0042 (10)	−0.0060 (8)
O5	0.0372 (8)	0.0272 (7)	0.0521 (9)	−0.0040 (6)	0.0057 (7)	0.0061 (7)
O6	0.0422 (8)	0.0266 (7)	0.0437 (8)	0.0026 (6)	0.0061 (7)	0.0000 (6)
O7	0.0819 (17)	0.0595 (14)	0.155 (3)	−0.0138 (12)	0.0739 (19)	−0.0093 (16)
O8	0.0713 (15)	0.0345 (10)	0.141 (2)	−0.0190 (10)	0.0299 (15)	0.0046 (13)
N1	0.0528 (11)	0.0276 (9)	0.0451 (11)	0.0108 (8)	−0.0003 (9)	0.0091 (8)
N2	0.0436 (11)	0.0353 (10)	0.0650 (14)	−0.0075 (9)	0.0087 (10)	0.0070 (10)
N3	0.0325 (8)	0.0287 (8)	0.0325 (8)	0.0070 (7)	0.0044 (7)	0.0091 (7)
N4	0.136 (3)	0.085 (2)	0.103 (3)	−0.028 (2)	−0.077 (2)	0.050 (2)
N5	0.0488 (10)	0.0272 (8)	0.0279 (8)	0.0093 (7)	0.0024 (7)	0.0072 (7)
N6	0.0717 (16)	0.0507 (14)	0.0597 (15)	−0.0003 (12)	−0.0179 (13)	0.0112 (12)
C1	0.0396 (10)	0.0240 (9)	0.0312 (9)	0.0086 (8)	0.0033 (8)	0.0072 (7)
C2	0.0361 (10)	0.0269 (9)	0.0346 (10)	0.0122 (8)	0.0055 (8)	0.0093 (8)
C3	0.0373 (10)	0.0282 (9)	0.0332 (10)	0.0105 (8)	0.0056 (8)	0.0092 (8)
C4	0.0411 (11)	0.0259 (9)	0.0348 (10)	0.0110 (8)	0.0006 (8)	0.0081 (8)
C5	0.0548 (14)	0.0371 (11)	0.0464 (13)	0.0196 (10)	0.0167 (11)	0.0048 (10)
C6	0.0565 (15)	0.0471 (14)	0.0628 (16)	0.0197 (12)	0.0313 (13)	0.0136 (12)
C7	0.0437 (12)	0.0347 (11)	0.0540 (14)	0.0086 (9)	0.0183 (10)	0.0098 (10)
C8	0.0323 (9)	0.0236 (9)	0.0298 (9)	0.0016 (7)	−0.0040 (7)	0.0055 (7)
C9	0.0303 (9)	0.0221 (8)	0.0321 (9)	0.0004 (7)	−0.0020 (7)	0.0045 (7)
C10	0.0331 (10)	0.0246 (9)	0.0387 (11)	0.0027 (7)	0.0041 (8)	0.0047 (8)
C11	0.0321 (10)	0.0261 (9)	0.0452 (12)	−0.0034 (8)	−0.0006 (9)	0.0071 (8)
C12	0.0485 (13)	0.0230 (10)	0.0587 (15)	0.0004 (9)	0.0032 (11)	−0.0023 (10)
C13	0.0547 (15)	0.0342 (12)	0.0656 (17)	0.0061 (11)	0.0202 (13)	−0.0062 (11)
C14	0.0387 (11)	0.0332 (11)	0.0509 (13)	−0.0005 (9)	0.0118 (10)	0.0007 (10)
C15	0.0334 (10)	0.0317 (10)	0.0388 (11)	0.0042 (8)	0.0001 (8)	0.0128 (8)
C16	0.0376 (11)	0.0411 (12)	0.0431 (12)	0.0086 (9)	−0.0002 (9)	0.0162 (10)
C17	0.0519 (14)	0.0486 (13)	0.0483 (13)	0.0137 (11)	0.0059 (11)	0.0269 (11)
C18	0.0567 (15)	0.0351 (12)	0.0568 (15)	0.0034 (10)	0.0089 (12)	0.0222 (11)
C19	0.0432 (12)	0.0323 (10)	0.0411 (12)	0.0017 (9)	0.0021 (9)	0.0095 (9)
C20	0.0715 (19)	0.0570 (17)	0.0665 (19)	−0.0031 (14)	−0.0272 (16)	0.0329 (15)
C21	0.0454 (12)	0.0296 (10)	0.0336 (10)	0.0072 (9)	0.0035 (9)	0.0093 (8)
C22	0.0408 (11)	0.0361 (11)	0.0310 (10)	0.0106 (9)	0.0019 (8)	0.0070 (8)
C23	0.0601 (15)	0.0393 (12)	0.0379 (11)	0.0157 (11)	−0.0025 (10)	0.0148 (10)
C24	0.0765 (18)	0.0296 (11)	0.0472 (13)	0.0060 (11)	−0.0058 (12)	0.0155 (10)
C25	0.0605 (15)	0.0284 (10)	0.0369 (11)	0.0030 (10)	−0.0055 (10)	0.0075 (9)
C26	0.0533 (14)	0.0412 (12)	0.0430 (12)	0.0080 (11)	−0.0028 (11)	0.0123 (10)

### Geometric parameters (Å, º)

**Table:**

Cd1—O1	2.3017 (15)	C7—H7	0.9300
Cd1—O2	2.5072 (18)	C8—C9	1.511 (3)
Cd1—O5	2.5367 (16)	C9—C10	1.393 (3)
Cd1—O5^i^	2.4716 (16)	C9—C14	1.389 (3)
Cd1—O6	2.3264 (15)	C10—C11	1.387 (3)
Cd1—N3	2.3186 (17)	C10—H10	0.9300
Cd1—N5	2.3435 (17)	C11—N2	1.470 (3)
Cd1—C1	2.733 (2)	C11—C12	1.377 (3)
O1—C1	1.264 (3)	C12—C13	1.381 (4)
O5—Cd1^i^	2.4716 (16)	C12—H12	0.9300
O5—C8	1.256 (3)	C13—H13	0.9300
O6—C8	1.253 (3)	C14—C13	1.386 (3)
O8—N2	1.210 (3)	C14—H14	0.9300
N1—O3	1.220 (3)	C15—C16	1.384 (3)
N1—O4	1.219 (3)	C15—H15	0.9300
N1—C4	1.472 (3)	C16—C17	1.387 (3)
N2—O7	1.198 (3)	C16—C20	1.438 (4)
N3—C15	1.330 (3)	C17—H17	0.9300
N3—C19	1.339 (3)	C18—C17	1.381 (4)
N4—C20	1.127 (4)	C18—H18	0.9300
N5—C21	1.335 (3)	C19—C18	1.379 (3)
N5—C25	1.331 (3)	C19—H19	0.9300
C1—O2	1.241 (3)	C21—C22	1.387 (3)
C1—C2	1.508 (3)	C21—H21	0.9300
C2—C7	1.381 (3)	C22—C23	1.391 (3)
C3—C2	1.388 (3)	C22—C26	1.443 (3)
C3—C4	1.384 (3)	C23—C24	1.375 (3)
C3—H3	0.9300	C23—H23	0.9300
C4—C5	1.378 (3)	C24—H24	0.9300
C5—C6	1.382 (4)	C25—C24	1.380 (3)
C5—H5	0.9300	C25—H25	0.9300
C6—H6	0.9300	C26—N6	1.144 (3)
C7—C6	1.397 (3)		
			
O1—Cd1—O2	54.33 (5)	C4—C5—H5	120.8
O1—Cd1—O5	161.73 (6)	C6—C5—H5	120.8
O1—Cd1—O5^i^	85.39 (6)	C5—C6—C7	120.1 (2)
O1—Cd1—O6	144.74 (6)	C5—C6—H6	120.0
O1—Cd1—N3	88.63 (6)	C7—C6—H6	120.0
O1—Cd1—N5	87.92 (6)	C2—C7—C6	120.5 (2)
O1—Cd1—C1	27.40 (6)	C2—C7—H7	119.7
O2—Cd1—O5	143.94 (5)	C6—C7—H7	119.7
O2—Cd1—C1	26.95 (6)	O5—C8—C9	119.48 (19)
O5^i^—Cd1—O2	139.57 (5)	O6—C8—O5	122.10 (18)
O5^i^—Cd1—O5	76.40 (5)	O6—C8—C9	118.41 (18)
O5—Cd1—C1	170.87 (6)	C10—C9—C8	119.84 (19)
O5^i^—Cd1—C1	112.66 (6)	C14—C9—C8	120.56 (19)
O6—Cd1—O2	90.60 (5)	C14—C9—C10	119.60 (18)
O6—Cd1—O5	53.47 (5)	C9—C10—H10	121.0
O6—Cd1—O5^i^	129.14 (5)	C11—C10—C9	117.9 (2)
O6—Cd1—N5	89.46 (6)	C11—C10—H10	121.0
O6—Cd1—C1	117.41 (6)	C10—C11—N2	118.8 (2)
N3—Cd1—O2	93.62 (6)	C12—C11—N2	117.92 (19)
N3—Cd1—O5	89.29 (6)	C12—C11—C10	123.3 (2)
N3—Cd1—O5^i^	88.16 (6)	C11—C12—C13	118.0 (2)
N3—Cd1—O6	98.23 (6)	C11—C12—H12	121.0
N3—Cd1—N5	170.84 (6)	C13—C12—H12	121.0
N3—Cd1—C1	92.02 (6)	C12—C13—C14	120.3 (2)
N5—Cd1—O2	91.25 (6)	C12—C13—H13	119.8
N5—Cd1—O5	91.33 (6)	C14—C13—H13	119.8
N5—Cd1—O5^i^	83.11 (6)	C9—C14—H14	119.6
N5—Cd1—C1	88.81 (6)	C13—C14—C9	120.9 (2)
C1—O1—Cd1	95.65 (12)	C13—C14—H14	119.6
C1—O2—Cd1	86.70 (13)	N3—C15—C16	121.9 (2)
Cd1^i^—O5—Cd1	103.60 (5)	N3—C15—H15	119.0
C8—O5—Cd1^i^	165.65 (15)	C16—C15—H15	119.0
C8—O5—Cd1	87.27 (13)	C15—C16—C17	119.8 (2)
C8—O6—Cd1	97.16 (12)	C15—C16—C20	119.9 (2)
O3—N1—C4	118.33 (19)	C17—C16—C20	120.3 (2)
O4—N1—O3	123.3 (2)	C16—C17—H17	120.9
O4—N1—C4	118.4 (2)	C18—C17—C16	118.1 (2)
O7—N2—O8	122.4 (2)	C18—C17—H17	120.9
O7—N2—C11	119.0 (2)	C17—C18—H18	120.7
O8—N2—C11	118.6 (2)	C19—C18—C17	118.7 (2)
C15—N3—Cd1	120.77 (14)	C19—C18—H18	120.7
C15—N3—C19	118.26 (19)	N3—C19—C18	123.2 (2)
C19—N3—Cd1	120.93 (14)	N3—C19—H19	118.4
C21—N5—Cd1	121.21 (14)	C18—C19—H19	118.4
C25—N5—Cd1	121.09 (15)	N4—C20—C16	178.3 (5)
C25—N5—C21	117.68 (19)	N5—C21—C22	122.5 (2)
O1—C1—Cd1	56.96 (10)	N5—C21—H21	118.7
O1—C1—C2	116.79 (18)	C22—C21—H21	118.7
O2—C1—Cd1	66.35 (12)	C21—C22—C23	119.3 (2)
O2—C1—O1	123.24 (19)	C21—C22—C26	119.7 (2)
O2—C1—C2	120.0 (2)	C23—C22—C26	121.0 (2)
C2—C1—Cd1	172.89 (15)	C22—C23—H23	121.1
C3—C2—C1	118.64 (19)	C24—C23—C22	117.9 (2)
C7—C2—C1	121.51 (19)	C24—C23—H23	121.1
C7—C2—C3	119.80 (19)	C23—C24—C25	119.2 (2)
C2—C3—H3	120.6	C23—C24—H24	120.4
C4—C3—C2	118.7 (2)	C25—C24—H24	120.4
C4—C3—H3	120.6	N5—C25—C24	123.4 (2)
C3—C4—N1	118.3 (2)	N5—C25—H25	118.3
C5—C4—N1	119.2 (2)	C24—C25—H25	118.3
C5—C4—C3	122.4 (2)	N6—C26—C22	178.8 (3)
C4—C5—C6	118.5 (2)		
			
O2—Cd1—O1—C1	−1.60 (12)	Cd1—O1—C1—C2	−176.08 (15)
O5—Cd1—O1—C1	179.36 (16)	Cd1—O5—C8—O6	−0.7 (2)
O5^i^—Cd1—O1—C1	174.69 (14)	Cd1^i^—O5—C8—O6	−140.5 (5)
O6—Cd1—O1—C1	5.3 (2)	Cd1—O5—C8—C9	178.05 (16)
N3—Cd1—O1—C1	−97.04 (14)	Cd1^i^—O5—C8—C9	38.3 (7)
N5—Cd1—O1—C1	91.44 (14)	Cd1—O6—C8—O5	0.8 (2)
O1—Cd1—O2—C1	1.62 (12)	Cd1—O6—C8—C9	−177.99 (15)
O5—Cd1—O2—C1	−178.89 (11)	O3—N1—C4—C3	−3.3 (3)
O5^i^—Cd1—O2—C1	−4.09 (18)	O3—N1—C4—C5	178.5 (2)
O6—Cd1—O2—C1	−174.39 (13)	O4—N1—C4—C3	176.7 (2)
N3—Cd1—O2—C1	87.33 (13)	O4—N1—C4—C5	−1.5 (3)
N5—Cd1—O2—C1	−84.92 (14)	Cd1—N3—C15—C16	−178.18 (17)
O1—Cd1—O5—Cd1^i^	−4.8 (2)	C19—N3—C15—C16	−0.7 (3)
O1—Cd1—O5—C8	−175.30 (18)	Cd1—N3—C19—C18	177.4 (2)
O2—Cd1—O5—Cd1^i^	176.53 (8)	C15—N3—C19—C18	0.0 (4)
O2—Cd1—O5—C8	6.03 (18)	Cd1—N5—C21—C22	−178.82 (17)
O5^i^—Cd1—O5—Cd1^i^	0.0	C25—N5—C21—C22	−0.5 (3)
O5^i^—Cd1—O5—C8	−170.51 (16)	Cd1—N5—C25—C24	179.2 (2)
O6—Cd1—O5—Cd1^i^	170.93 (10)	C21—N5—C25—C24	0.8 (4)
O6—Cd1—O5—C8	0.42 (12)	O1—C1—O2—Cd1	−2.9 (2)
N3—Cd1—O5—Cd1^i^	−88.28 (7)	C2—C1—O2—Cd1	176.33 (17)
N3—Cd1—O5—C8	101.21 (13)	O1—C1—C2—C3	−15.8 (3)
N5—Cd1—O5—Cd1^i^	82.58 (7)	O1—C1—C2—C7	161.6 (2)
N5—Cd1—O5—C8	−87.92 (13)	O2—C1—C2—C3	164.9 (2)
O1—Cd1—O6—C8	177.25 (12)	O2—C1—C2—C7	−17.6 (3)
O2—Cd1—O6—C8	−177.13 (13)	C1—C2—C7—C6	−176.7 (2)
O5—Cd1—O6—C8	−0.42 (12)	C3—C2—C7—C6	0.7 (4)
O5^i^—Cd1—O6—C8	10.98 (16)	C4—C3—C2—C1	177.69 (18)
N3—Cd1—O6—C8	−83.38 (13)	C4—C3—C2—C7	0.2 (3)
N5—Cd1—O6—C8	91.63 (13)	C2—C3—C4—N1	−179.25 (18)
C1—Cd1—O6—C8	−179.99 (12)	C2—C3—C4—C5	−1.1 (3)
O1—Cd1—N3—C15	146.88 (17)	N1—C4—C5—C6	179.1 (2)
O1—Cd1—N3—C19	−30.50 (17)	C3—C4—C5—C6	0.9 (4)
O2—Cd1—N3—C15	92.75 (17)	C4—C5—C6—C7	0.1 (4)
O2—Cd1—N3—C19	−84.63 (17)	C2—C7—C6—C5	−0.9 (4)
O5—Cd1—N3—C15	−51.27 (16)	O5—C8—C9—C10	−176.4 (2)
O5^i^—Cd1—N3—C15	−127.69 (17)	O5—C8—C9—C14	3.1 (3)
O5—Cd1—N3—C19	131.34 (17)	O6—C8—C9—C10	2.4 (3)
O5^i^—Cd1—N3—C19	54.93 (17)	O6—C8—C9—C14	−178.1 (2)
O6—Cd1—N3—C15	1.62 (17)	C8—C9—C10—C11	178.96 (19)
O6—Cd1—N3—C19	−175.77 (17)	C14—C9—C10—C11	−0.6 (3)
C1—Cd1—N3—C15	119.69 (17)	C8—C9—C14—C13	179.8 (2)
C1—Cd1—N3—C19	−57.69 (17)	C10—C9—C14—C13	−0.6 (4)
O1—Cd1—N5—C21	25.91 (17)	C9—C10—C11—N2	−179.8 (2)
O1—Cd1—N5—C25	−152.4 (2)	C9—C10—C11—C12	1.4 (4)
O2—Cd1—N5—C21	80.15 (18)	C10—C11—N2—O7	−7.6 (4)
O2—Cd1—N5—C25	−98.16 (19)	C10—C11—N2—O8	173.3 (3)
O5—Cd1—N5—C21	−135.83 (17)	C12—C11—N2—O7	171.3 (3)
O5—Cd1—N5—C25	45.86 (19)	C12—C11—N2—O8	−7.7 (4)
O5^i^—Cd1—N5—C25	122.0 (2)	N2—C11—C12—C13	−179.7 (3)
O5^i^—Cd1—N5—C21	−59.69 (17)	C10—C11—C12—C13	−0.9 (4)
O6—Cd1—N5—C25	−7.57 (19)	C11—C12—C13—C14	−0.4 (5)
O6—Cd1—N5—C21	170.74 (18)	C9—C14—C13—C12	1.2 (5)
C1—Cd1—N5—C21	53.30 (18)	N3—C15—C16—C17	0.9 (4)
C1—Cd1—N5—C25	−125.0 (2)	N3—C15—C16—C20	−177.6 (3)
O1—Cd1—C1—O2	−177.1 (2)	C15—C16—C17—C18	−0.2 (4)
O2—Cd1—C1—O1	177.1 (2)	C20—C16—C17—C18	178.2 (3)
O5^i^—Cd1—C1—O1	−5.74 (15)	C19—C18—C17—C16	−0.4 (4)
O5^i^—Cd1—C1—O2	177.13 (13)	N3—C19—C18—C17	0.6 (4)
O6—Cd1—C1—O1	−176.54 (13)	N5—C21—C22—C23	−0.3 (4)
O6—Cd1—C1—O2	6.33 (15)	N5—C21—C22—C26	178.2 (2)
N3—Cd1—C1—O1	83.11 (14)	C21—C22—C23—C24	0.6 (4)
N3—Cd1—C1—O2	−94.02 (14)	C26—C22—C23—C24	−177.8 (3)
N5—Cd1—C1—O1	−87.76 (14)	C22—C23—C24—C25	−0.3 (4)
N5—Cd1—C1—O2	95.10 (14)	N5—C25—C24—C23	−0.5 (5)
Cd1—O1—C1—O2	3.1 (2)		

Symmetry code: (i) −*x*, −*y*+1, −*z*+1.

### Hydrogen-bond geometry (Å, º)

**Table:**

*D*—H···*A*	*D*—H	H···*A*	*D*···*A*	*D*—H···*A*
C14—H14···O1^i^	0.93	2.20	3.108 (3)	167
C15—H15···O2^ii^	0.93	2.32	3.111 (3)	143
C23—H23···N4^iii^	0.93	2.38	3.236 (5)	154
C25—H25···O6	0.93	2.58	3.242 (3)	128
C10—H10···*Cg*3^ii^	0.93	3.26	4.186 (3)	176

Symmetry codes: (i) −*x*, −*y*+1, −*z*+1; (ii) −*x*+1, −*y*+1, −*z*+1; (iii) *x*−1, *y*, *z*−1.
